# Multiple soft tissue aneurysmal cysts: An occurrence after resection of primary aneurysmal bone cyst of fibula

**DOI:** 10.4103/0019-5413.33693

**Published:** 2007

**Authors:** P Karkuzhali, Mahuya Bhattacharyya, P Sumitha

**Affiliations:** Institute of Pathology, Madras Medical College, Chennai - 600 003, Tamil Nadu, India

**Keywords:** Aneurysmal cyst, fibula, multiple, secondary to resection of primary aneurysmal bone cyst, soft tissue

## Abstract

We report a case of multiple extraosseous aneurysmal cysts occurring in the muscle and subcutaneous plane of postero-lateral aspects of the upper right leg. They appeared about 15 months after resection of aneurysmal bone cyst of the upper end of the fibula. They varied in size from 2 cm to 5 cm. Radiologically they were well-defined lesions with central septate areas surrounded by a rim of calcification. Histologically they showed central cystic spaces separated by septa consisting of fibroblasts, osteoclast type of giant cells and reactive woven bone. Thus they showed histological similarity with aneurysmal bone cysts, but did not show any connection with the bone. Only very few examples of aneurysmal cysts of soft tissue had been described in the past one decade and they were reported in various locations including rare sites such as arterial wall and larynx. Recent cytogenetic analyses have shown abnormalities involving 17p11-13 and/or 16q22 in both osseous and extraosseous aneurysmal cysts indicating its probable neoplastic nature. Our case had unique features like multiplicity and occurrence after resection of primary aneurysmal bone cyst of the underlying bone.

Aneurysmal cyst of soft tissue is a recently recognized entity which shares certain clinical, radiological and histological features with myositis ossificans. However, the typical histological features are that of aneurysmal bone cyst and consist of central multiple cystic cavities separated by thin and thick-walled septa consisting of fibroblasts, osteoclast type of giant cells and spicules of bone. It is surrounded by a rim of reactive bone in varying stages of maturation. Initially it was thought to be a nonneoplastic condition, but recently, reproducible clonal chromosomal abnormalities have been described in both osseous and extraosseous aneurysmal bone cysts suggesting its probable neoplastic nature.^1^–^3^ Aneurysmal cyst of soft tissue is a rare entity and only 16 cases have been described in the English literature so far. They have been described to occur in muscle and subcutaneous planes in various locations and in rare sites such as arterial wall^4^ and larynx.^5^ To the best of our knowledge it may be the first case of multiple aneurysmal cysts occurring after resection of a primary aneurysmal bone cyst of the underlying bone.

## CASE REPORT

A 23-year-old male presented with complaints of multiple swellings over the posterior and lateral aspects of the right leg of five months duration. He had a swelling at the same site for which he was operated about 20 months prior to present admission. It was an osteolytic lesion in the upper end of the fibula for which resection of the head and neck of fibula was done. The lesion was histologically diagnosed as aneurysmal bone cyst. No soft tissue component was evident at that time.

On examination, multiple swellings varying in size from 2×1 cm to 4×2 cm were seen involving the posterior and lateral aspect of the upper third of the right leg. They were firm to hard in consistency, non tender and their mobility was restricted partially. Routine laboratory tests were within normal limits. The serum alkaline phosphatase, phosphorus, calcium and uric acid levels were normal.

**Figure 1:**
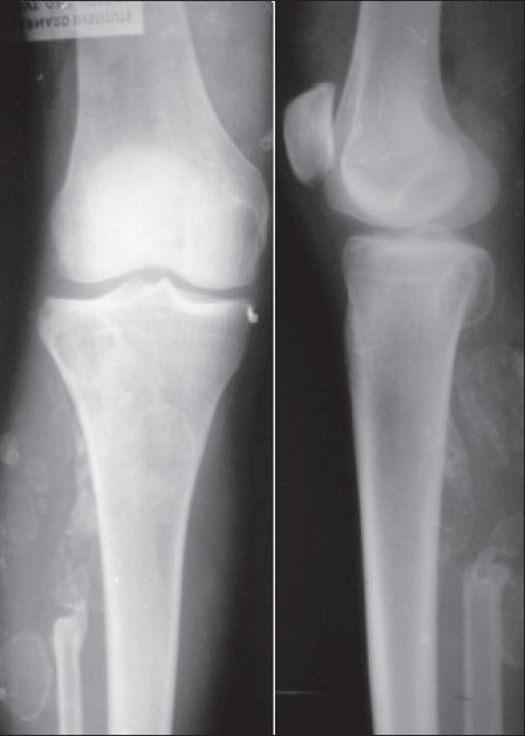
Plain X-ray (AP and lateral view) showing multiple radio opaque septate rim calcified lesions in soft tissue.

Plain X-rays [Figure 1] showed multiple well-defined, radio opaque, septate lesions with calcification of rims in the muscular plane of the upper right leg. Upper part of the fibula was absent. An expansile lytic lesion was seen in the postero-lateral aspect of the epimetaphyseal region of the upper end of tibia. The computerized tomography (CT) scan showed multiple hyperdense lesions within the gastrocnemius and soleus muscles and also in the subcutaneous plane. A few lesions showed rim calcification with central soft tissue component. During the operation, the masses were seen within and near the gastrocnemius and soleus muscles and in the subcutaneous plane. The largest three soft tissue lesions were excised in toto.

### Pathology

**Figure 2:**
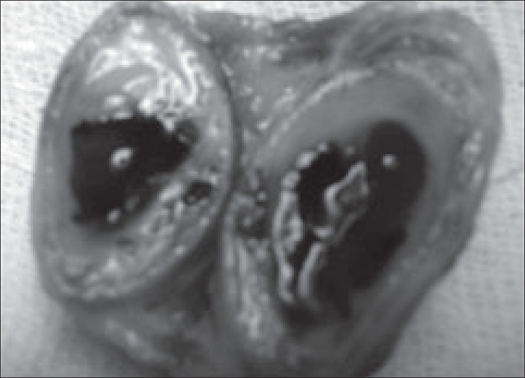
Excised cystic mass showing central cystic blood filled cavity with septa surrounded by a rim of bone.

All the three excised specimens were well-circumscribed, ovoid with smooth surfaces. They measured 4×2×2 cm, 3×2×1 cm and 2×1×1 cm. On sectioning central large cystic cavities containing reddish and bloody viscous material traversed by a few septa and surrounded by a rim of bone were seen [Figure 2].

**Figure 3:**
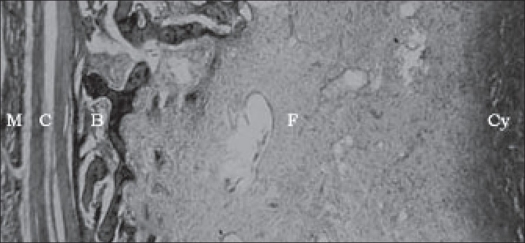
Microscopic features. M - Muscle, C - capsule, B - bone, F - Fibromyxoid stroma and Cy - cystic blood filled space without any lining endothelium (H and E, ×10)

**Figure 4:**
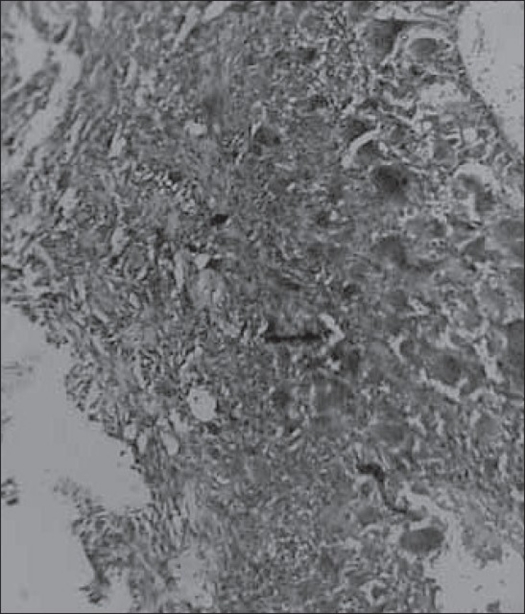
Cellular area in septum showing plump fibroblasts and osteoclast type of giant cells (H and E, ×40)

Microscopically, central solitary or multiple cystic spaces were seen that were separated and surrounded by fibromyxoid stroma and reactive bone in varying stages of maturation. A thick condensation of fibrous tissue was seen encircling the lesion completely. The whole mass was seen embedded in muscle tissue. The central cystic spaces contained blood and did not show any endothelial lining [Figure 3]. The septa consisted of haphazardly arranged spindle-shaped fibroblasts admixed with lymphocytes, macrophages and few osteoclast type of giant cells. Some foci were cellular with plump fibroblasts and osteoclast type of giant cells. No cellular or nuclear atypia was seen [Figure 4]. Central cystic spaces were completely encircled by thin and thick trabeculae of osteoid and heavily mineralized woven bone with or without rimming by osteoblasts. One specimen showed plenty of heavily mineralized ‘blue bone’, characteristic of aneurysmal bone cyst.

## DISCUSSION

Most of the bone tumors such as chondroma,^6^ chondroblastoma,^7^ giant cell tumors,^8^–^9^ chondrosarcoma and osteosarcoma^6^ have already been described in soft tissues. But the extraosseous counterpart of aneurysmal cysts has been reported in the literature only in the past 10 years. The first two cases of aneurysmal cysts of soft tissue were probably reported by Salm and Sissons in 1972.^8^ While reporting on giant cell tumors of soft tissues, they mentioned two cases which had distinct histological features similar to aneurysmal bone cysts and designated them as “vascular cystic tumors of soft tissue”. Amir *et al.* reported a soft tissue lesion in the left groin of a seven-year-old boy which they considered as aneurysmal cyst-like changes in myositis ossificans.^10^ Since then about 14 cases (including this study) of extraosseous aneurysmal cysts have been reported in various locations.

A review of the literature shows that the age of these patients ranged from seven years to 73 years. There was no sex predilection (M: F= 9:8). They generally presented as slow-growing masses. No history of trauma was obtained in any of these cases. The tumors occurred within and near the muscle of the upper and lower extremities, shoulder, retroclavicular region, hip, groin, pelvic cavity and abdominal wall. The rarest sites included the arterial wall of the bifurcation of the common carotid artery and larynx [Table 1]. The size of these tumors ranged from 2 cm to 9 cm. Radiologically these tumors were well-demarcated lesions with central soft tissue component and peripheral rim of calcification and bone formation. They did not show any connection with the adjacent bone. Histologically they were well-defined lesions with central cystic spaces traversed by septa composed of fibroblasts, osteoclast type of giant cells and spicules of bone. Fibromyxoid stroma which is a characteristic feature of aneurysmal bone cyst also shows presence of chronic inflammatory cells including macrophages and lymphocytes. The lesions are completely encircled by trabeculae of osteoid, woven bone and even lamellar bone. According to the information available the tumors do not recur or metastasize if the tumors are completely excised. Excision appears to be the adequate treatment.

**Table 1:** Soft tissue aneurysmal cysts

Case No.	Source and year	Age/sex	Location
1 and 2	Salm *et al.*,^8^	32/m	Thigh
		45/m	Abdominal wall
3	Amir *et al.*,^10^	15/f	Groin
4	Petrik *et al.*,^4^	7/m	(Lt) common carotid artery
5	Rodriquez-Peralto *et al.*,^13^	20/f	(Lt) shoulder
6	Lopez-Barea *et al.*,^14^	57/f	Arm
7	Ricconi R *et al.*,^15^	73/m	(Lt) hip
8	Shannon *et al.*,^11^	29/f	Retroclavicular
9	Samura H *et al.*,^16^	51/f	Pelvic cavity
10	Della Libera *et al.*,^5^	22/m	Larynx
		8/m	(Rt) shoulder
		29/f	(Rt) groin
11-15	Nielsen GP *et al.*,^17^	37/f	Upper arm
		28/m	(Lt) deltoid
		30/f	Thigh
16	Wang *et al.*,^18^	21/m	(Rt) gluteus medius
17	Our study	23/m	(Rt) leg

The differential diagnosis of soft tissue aneurysmal cysts include nodular fasciitis with osteoclast type of giant cells, giant cell tumors of soft tissues, ossifying fibromyxoid tumor, extraskeletal osteogenic sarcoma and myositis ossificans. The septa may show histological features similar to nodular fasciitis with osteoclast type of giant cells. In fact a diagnosis of nodular fasciitis was initially made in one of the published cases of soft tissue aneurysmal cysts.^11^ However, prominent cystic changes and peripheral rim of bone formation are not features of nodular fasciitis. Soft tissue giant cell tumor may show cystic change. But the cysts are not as prominent and the solid areas with characteristic cellular changes of giant cell tumor are lacking in soft tissue aneurysmal cyst.^8^–^9^ Ossifying fibromyxoid tumor may show a peripheral shell of mature bone, but it shows a lobular growth pattern and the cells are arranged in nests and cords in a myxoid or hyaline stroma.^6^ Cystic change with septa containing fibroblasts and osteoclast type of giant cells are not features of ossifying fibromyxoid tumor. Extraskeletal osteogenic sarcoma of telengiectatic type, which is a very rare lesion may mimic aneurysmal cyst to some extent.^12^ However, closer examination for cytological atypia helps to distinguish the former from the latter. Soft tissue aneurysmal cyst shares several morphologic features with myositis ossificans. The distinct features of myositis ossificans include its solid nature and distinct zonation. The orderly maturation of fibroblasts into mature bone which is characteristic of myositis ossificans is not seen regularly in aneurysmal cyst of soft tissues.

The histogenesis of this lesion is not yet clear. It had been suggested that myositis ossificans and soft tissue aneurysmal cyst might represent different reaction patterns to perceived or unperceived injury to soft tissue or that soft tissue aneurysmal cyst is a cystic variant of myositis ossificans. The latter is substantiated by the location of soft tissue aneurymal cysts and its radiological and histological features. Furthermore, an aneurysmal cyst of soft tissue has been shown to evolve into a cystic lesion from an initial solid lesion, by sequential MRI imaging studies.^11^ A vague zonation is seen in aneurysmal cysts too, suggesting that aneurysmal cysts might be an advanced cystic lesion that has evolved from preexisting myositis ossificans. But recent cytogenetic analyses^1^–^3^ have shown translocations involving 16q22 and 17p13 indicating its probable neoplastic nature. More number of cytogenetic studies in myositis ossificans and soft tissue aneurysmal cysts might help in understanding the histogenesis of these lesions.

This report is probably the first case of multiple aneurysmal cysts occurring after resection of primary aneurysmal bone cyst of the upper end of the fibula. This finding along with the histological feature of complete encapsulation in all masses suggests a neoplastic nature. The association with the translocations involving 16q22 and 17p13 also is an indication of its neoplastic nature. The case is reported for its rarity and to create an awareness of several primary bone lesions occurring in soft tissues exclusively or in combination with underlying bone lesions.
